# Comparing oncologic and surgical outcomes of robotic and laparoscopic distal pancreatectomy: a propensity-matched analysis

**DOI:** 10.1007/s00464-024-11147-5

**Published:** 2024-08-12

**Authors:** Jenny H. Chang, Chase Wehrle, Kimberly Woo, Robert Naples, Kathryn A. Stackhouse, Fadi Dahdaleh, Daniel Joyce, Robert Simon, Toms Augustin, R. Matthew Walsh, Samer A. Naffouje

**Affiliations:** 1 Department of General Surgery, Cleveland Clinic Foundation, 18101 Lorain Avenue, Cleveland, OH 44111 USA; 2 Department of Surgical Oncology, Edward-Elmhurst Health, Elmhurst, IL USA

**Keywords:** Robotic distal pancreatectomy, Laparoscopic distal pancreatectomy, National cancer database

## Abstract

**Background:**

The frequency of minimally invasive distal pancreatectomy is gradually exceeding that of the open approach. Our study aims to compare short-term outcomes of robotic (RDP) and laparoscopic (LDP) distal pancreatectomies for pancreatic ductal adenocarcinoma (PDAC) using a national database.

**Methods:**

The National Cancer Database was utilized to identify patients with PDAC who underwent distal pancreatectomy from 2010–2020. Short-term technical and oncologic outcomes such as margin status and nodal harvest were included. Propensity-score matching (PSM) was performed comparing LDP and RDP cohorts. Multivariate logistic-regression models were then used to assess the impact of institutional volume on the MIDP surgical and technical oncologic outcomes.

**Results:**

1537 patients underwent MIDP with curative intent. Most cases were laparoscopic (74.4%, *n* = 1144), with a gradual increase in robotic utilization, from 8.7% in 2010 to 32.0% of MIDP cases ten years later. For PSM, 698 LDP patients were matched with 349 RDP. The odds of conversion to an open case were 58% less in RDP (12.6%) compared to LDP (25.5%) with no statistically significant difference in technical oncologic results. There was no difference in length of stay (OR = 1.0[0.7–1.4]), 30-day mortality (OR = 0.5[0.2–2.0]) or 90-day mortality (OR = 1.1[0.5–2.4]) between RDP and LDP, although there was a higher 30-day readmission rate with RDP (OR = 1.71[1.1–2.7]). There were statistically significant differences in technical oncologic outcomes (nodal harvest, margin status, initiation of adjuvant therapy) based on MIDP volume quartiles.

**Conclusion:**

Laparoscopic and robotic distal pancreatectomy have similar peri- and post-operative surgical and oncologic outcomes, with a higher rate of conversion to open in the laparoscopic cohort.

Minimally invasive techniques of both laparoscopic and robotic approaches to pancreatectomy have increased in frequency since the laparoscopic pancreatectomy was first reported in 1994 [1]. In particular, minimally invasive distal pancreatectomy (MIDP) has been rapidly expanding in surgeon preference and utilization, and is now performed more frequently than the open approach [2, 3]. Multiple randomized controlled trials, such as LEOPARD and LAPOP, have demonstrated superior outcomes in functional recovery and length of stay of MIDP as compared to open distal pancreatectomy (ODP), with comparable morbidity [4, 5]. Regarding technical oncologic outcomes for pancreas cancer, the recent DIPLOMA randomized control trial demonstrated that the R0 resection rate and lymph node yield of MIDP to ODP were comparable [6].

However, there has been no randomized control trials directly comparing the two minimally invasive approaches and specific differences in technical oncologic outcomes between the two MIDP modalities are also not yet elucidated. It is theorized that the robotic platform could provide an enhanced visualization and dexterity for oncologic benefits such as higher rates of negative margin resections and improved lymph node harvest [7]. Nevertheless, many retrospective studies are limited by a conglomeration of benign and malignant pancreatic lesions, selection bias by surgeon, and variation in both the surgeon and institution volume in pancreatic surgery.

Our study aims to compare RDP and LDP using a national database of hospital-based cohorts over a course of ten years, focusing on short-term technical outcomes and oncologic metrics specific for pancreatic ductal adenocarcinoma. Considering increasing utilization of MIDP, we then compare these outcomes of MIDP by institution volume.

## Methods

This is a retrospective study utilizing the National Cancer Database (NCDB), a nationwide database which captures hospital registry data collected in more than 1500 American College of Surgeons Commission on Cancer-accredited facilities [8]. The NCDB captures oncologic and limited operative variables for site-specific treatment of the primary cancer. For this study, we used the 2010–2020 database. Patients who were ≥ 18 years of age, had a diagnosis of pancreatic ductal adenocarcinoma (PDAC), and underwent a distal pancreatectomy of any approach were included. Those less than 18 years of age, underwent distal pancreatectomy (±splenectomy) with another procedure, underwent a diagnostic laparoscopy followed by ODP, or those who did not have a diagnosis of PDAC were excluded from the analysis.

Queried variables included demographic and limited clinical variables including the Charlson comorbidity score (CCI). Oncologic information included tumor (T) stage and neoadjuvant and adjuvant therapies. Surgical information and outcomes included initial approach, conversion rate, length of stay, unplanned readmission, and 30- and 90- day mortality as collected in the NCDB. Specific surgical oncologic outcomes analyzed included margin status and number of lymph node harvest and initiation of adjuvant therapy. Finally, center-level data were queried, including facility-type, facility location (metropolitan, urban, rural), and annual center volume of MIDP.

Descriptive statistics were used to summarize patient demographics, oncologic information, and surgical variables. Categorical variables were presented as frequencies and percentages. Continuous variables were presented as means with standard deviations or medians with interquartile range as appropriate. Propensity-score matching (PSM) was then performed to account for potential confounding variables between the LDP and RDP cohorts. Matching was performed between groups in a 2:1 fashion. PSM utilized the nearest neighbor method per propensity scores with a caliper width of 0.05. Groups were matched on year of procedure, age, sex, race, CCI, T stage (T1–T3), and neoadjuvant therapy (chemotherapy and radiation). To validate the matching results, the effect size between the matched groups using Theil’s U test for categorical variables and Cohen’s d test (standard difference) for continuous variables was calculated. Odds ratios with 95% confidence interval were calculated for the surgical and technical oncologic outcomes. Finally, multivariate logistic-regression models were used to assess the impact of institutional volume quartiles on the MIDP surgical and technical oncologic outcomes.

SPSS v29.0 (Armonk, NY) was used for this statistical analysis and *α* < 0.05 was set as a threshold for statistical significance throughout the study.

## Results

The NCDB database included 22,172 patients who underwent distal pancreatic resection between 2010 to 2020. Of these, 12,635 (57.0%) were open, 6708 (30.3%) were laparoscopic, and 2829 were (12.8%) robotic. After application of our inclusion and exclusion criteria, a final cohort of 1537 patients who have undergone MIDP with curative intent were selected.

**Fig. 1:**
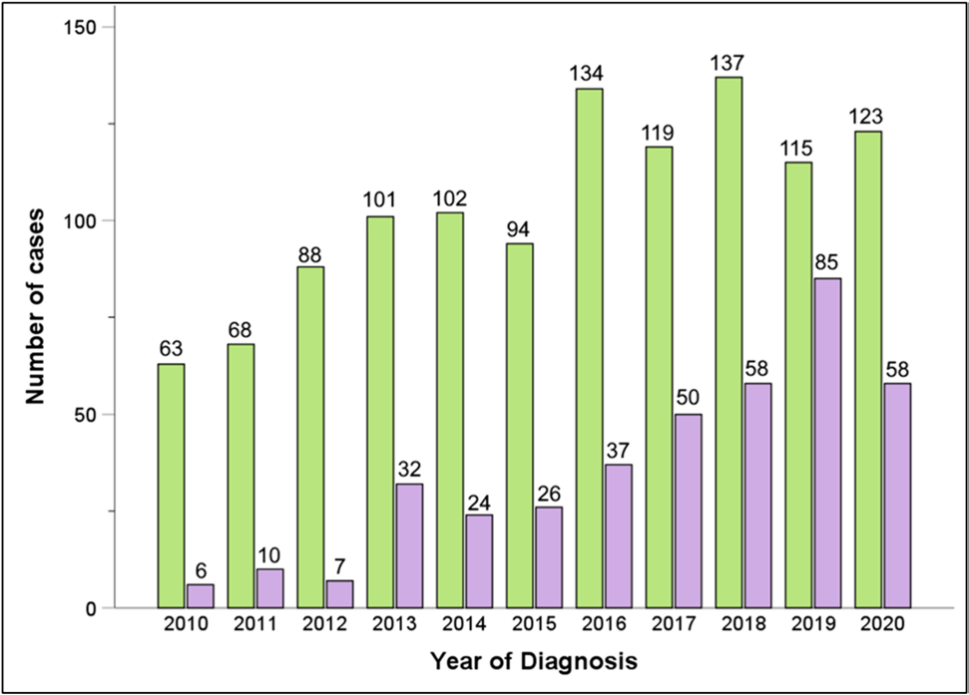
Chronological trends of LDP and RDP during the study period between 2010 and 2020. *LDP* Laparoscopic Distal Pancreatectomy, *RDP* Robotic Distal Pancreatectomy

Overall, most of these MIDP cases were performed laparoscopically (74.4%, *n* = 1144), and the remaining 25.6% (*n* = 393) was performed robotically. However, chronological trends show a gradual increase of the robotic platform utilization, from 8.7% of MIDP cases in 2010 to nearly one third (32.0%) of MIDP cases ten years later in 2020 (Fig. 1). Most (53.4%, *n* = 820) MIDP cases were performed at an academic or research institution in a metropolitan location (81.3%, *n* = 1250). The median number [IQR] of MIDP performed at participating institutions was 3 [1–5] per year.

Of these 1537 patients, 21.0% (*n* = 323) were converted to open. Surgical oncologic outcomes of MIDP include a 12.7% positive margin rate and a mean of 15.4 ± 10.1 nodes retrieved. The mean length of stay was 6.2 days, with a 7.7% unplanned 30-day readmission rate. The 30-day and 90-day mortality rates were 1.4% (*n* = 22) and 2.9% (*n* = 44), respectively, as demonstrated in Table 1.

**Table 1:** Demographic and clinical characteristics of selected patients with pancreatic adenocarcinoma in the head of pancreas who underwent laparoscopic or robotic DP with curative intent between 2010–2020

N		1537
Age	Mean ± SD, median	68.1 ± 10.99, 69
Sex	Male	740 (48.1%)
	Female	797 (51.9%)
Race	White	1220 (79.4%)
	Black	182 (11.8%)
	Other	135 (8.8%)
Charlson score	0	883 (79.4%)
	1	429 (27.9%)
	2	122 (7.9%)
	3	103 (6.7%)
T stage	T1	319 (20.8%)
	T2	401 (26.1%)
	T3	817 (53.2%)
Neoadjuvant therapy	Chemotherapy	298 (19.4%)
	Radiation	76 (4.9%)
Facility type	CCC	15 (1.0%)
	CCCP	359 (23.4%)
	A/RP	820 (53.4%)
	INCP	323 (21.0%)
	Not reported	20 (1.3%)
Annual DP center volume	Median [IQR]	5 [3–11]
Annual MIDP center volume	Median [IQR]	3 [1–5]
Area	Metropolitan	1250 (81.3%)
	Urban	183 (11.9%)
	Rural	31 (2.0%)
	Not reported	73 (4.7%)
Approach	Laparoscopic DP	1144 (74.4%)
	Robotic DP	393 (25.6%)
Conversion	No	1214 (79.0%)
	Yes	323 (21.0%)
Margins	Negative	1327 (86.3%)
	Positive	210 (13.7%)
Examined nodes	Mean ± SD, median	15.4 ± 10.1, 14
Length of stay	Mean ± SD, median	6.2 ± 4.7, 5
Unplanned 30-day readmission		119 (7.7%)
Mortality	30-day	22 (1.4%)
	90-day	44 (2.9%)
Adjuvant systemic therapy		874 (56.9%)

*A/RP* Academic/Research Program, *CCC* Community Cancer Program, *CCCP* Comprehensive Community Cancer Program, *DP* Distal Pancreatectomy, *INCP* Integrated Network Cancer Program, *IQR* Interquartile Range, *SD* Standard Deviation

**Fig. 2:**
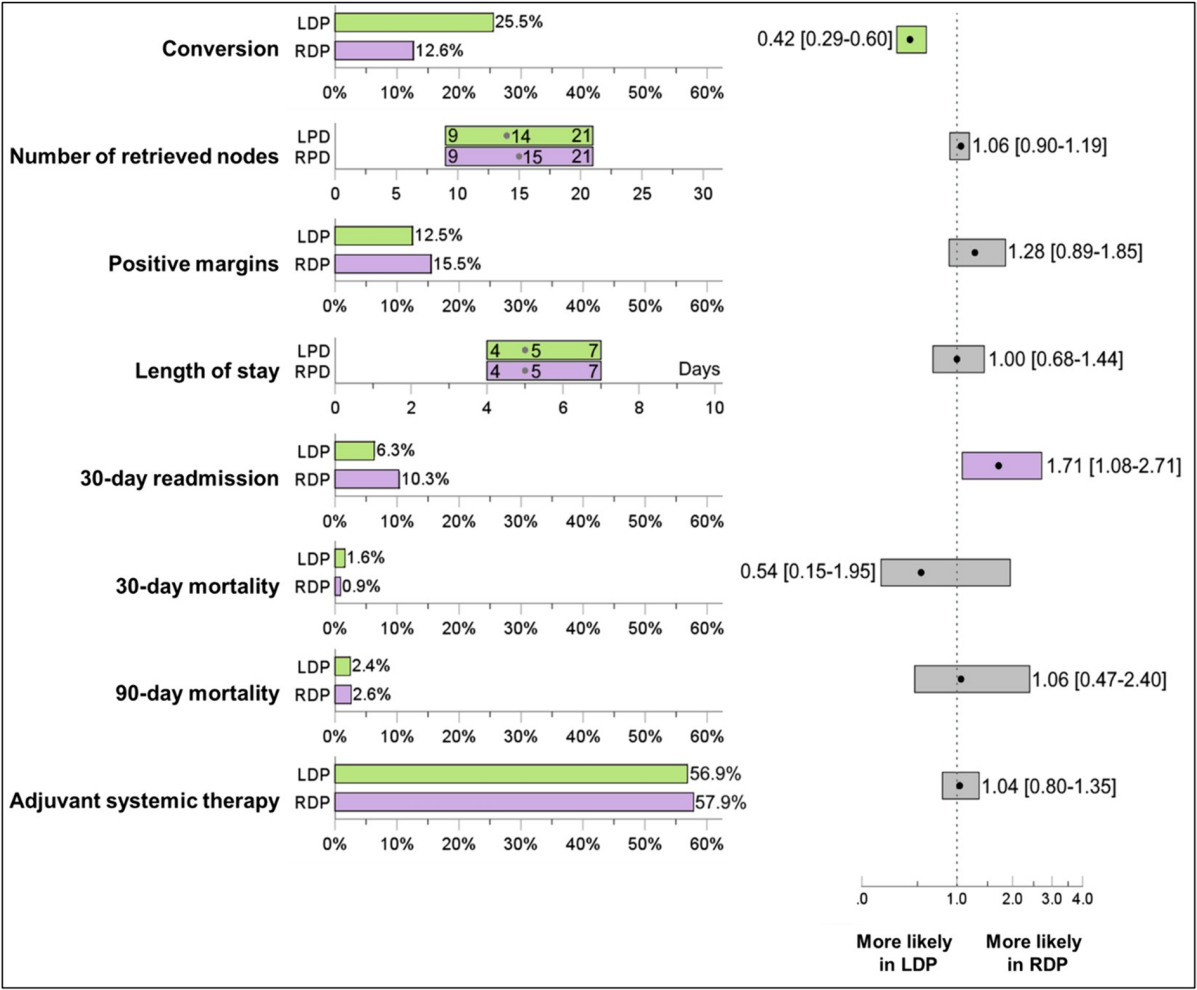
Comparison of short-term quality outcomes between the matched LDP and RDP groups. *LDP* Laparoscopic Distal Pancreatectomy, *RDP* Robotic Distal Pancreatectomy

Comparing baseline demographics of LDP and RDP groups, differences in the distribution of year of procedure (*p* < 0.01) were noted, with the increased frequency of both procedure approaches in later years of the study period. Clinical differences in baseline between the LDP and RDP groups also included T stage (*p* < 0.01) and use of neoadjuvant chemotherapy (*p* < 0.01) were noted. More patients in the LDP group had larger tumors (55.3% with T3 in LDP group) than the RDP group (46.8%). Propensity-score matching (PSM) was performed in a 2:1 ratio, matching 698 patients from the LDP to 349 patients in the RDP groups, which resolved the differences in the two groups. Table 2 demonstrates the comparison between the unmatched and 2:1 matched LDP and RDP cohorts. Comparison of these matched cohorts, as shown in Fig. 2, demonstrate that the odds of conversion to an open case is 58% less likely in an RDP compared to a LDP (OR = 0.42 [0.3–0.6]). However, there is no significant difference in the mean number of retrieved nodes between LDP (14 [9–21] nodes) or RDP (15 [9–21] nodes), or in the rate of resection with positive margins (12.5% vs. 15.5%, OR = 1.3 [0.9–1.89]). There was also no difference in length of stay, 30-day mortality and 90-day mortality rates, although there was higher odds of 30-day readmission with the RDP group (6.3% vs. 10.3%, OR = 1.7 [1.1–2.7]). Finally, there is no significant difference in adjuvant systemic therapy initiation as well (56.9% for LDP versus 57.9% for RDP, OR = 1.04 [0.8–1.4]).

**Table 2:** Comparison of baseline characteristics between the unmatched and 2:1 matched LDP and RDP patients

	LDP	RDP	ES^b^	p	LDP	RDP	ES^b^	p
N	1144	393			698	349		
Year of procedure			0.217	** < 0.001**^a^			0.002	0.181
2010	63 (5.5%)	6 (1.5%)			14 (2.0%)	5 (1.4%)		
2011	68 (5.9%)	10 (2.5%)			11 (1.6%)	10 (2.9%)		
2012	88 (7.7%)	7 (1.8%)			31 (4.4%)	7 (2.0%)		
2013	101 (8.8%)	32 (8.1%)			44 (6.3%)	32 (9.2%)		
2014	102 (8.9%)	24 (6.1%)			45 (6.4%)	24 (6.9%)		
2015	94 (8.2%)	26 (6.6%)			55 (7.9%)	26 (7.4%)		
2016	134 (11.7%)	37 (9.4%)			94 (13.5%)	37 (10.6%)		
2017	119 (10.4%)	50 (12.7%)			94 (13.5%)	49 (14.0%)		
2018	137 (12.0%)	58 (14.8%)			118 (16.9%)	52 (14.9%)		
2019	115 (10.1%)	85 (21.6%)			99 (14.2%)	64 (18.3%)		
2020	123 (10.8%)	58 (14.8%)			93 (13.3%)	43 (12.3%)		
Age	67.8 ± 11.2	68.7 ± 10.3	0.076	0.193	68.6 ± 10.5	68.7 ± 10.2	0.013	0.848
Sex			0.017	0.498			0.031	0.861
Male	545 (47.6%)	195 (49.6%)			344 (49.3%)	170 (48.7%)		
Female	599 (52.4%)	198 (50.4%)			354 (50.7%)	179 (51.3%)		
Race			0.025	0.503			0.002	0.438
White	916 (80.1%)	304 (77.4%)			556 (79.7%)	274 (78.5%)		
Black	130 (11.4%)	52 (13.2%)			73 (10.5%)	45 (12.9%)		
Other	98 (8.6%)	37 (9.4%)			69 (9.9%)	30 (8.6%)		
Charlson score			0.024	0.728			0.010	0.828
0	651 (56.9%)	232 (59.0%)			408 (58.5%)	198 (56.7%)		
1	322 (28.1%)	107 (27.2%)			190 (27.2%)	98 (28.1%)		
2	90 (7.9%)	32 (8.1%)			52 (7.4%)	31 (8.9%)		
3	81 (7.1%)	22 (5.6%)			48 (6.9%)	22 (6.3%)		
T stage			0.166	**0.004**^a^			0.028	0.496
T1	236 (20.6%)	83 (21.1%)			158 (22.6%)	72 (20.6%)		
T2	275 (24.0%)	126 (32.1%)			187 (26.8%)	105 (30.1%)		
T3	633 (55.3%)	184 (46.8%)			353 (50.6%)	172 (49.3%)		
Neoadjuvant therapy								
Chemotherapy	198 (17.3%)	100 (25.4%)	0.090	** < 0.001**^a^	131 (18.8%)	73 (20.9%)	0.024	0.410
Radiation	59 (5.2%)	17 (4.3%)	0.019	0.512	33 (4.7%)	16 (4.6%)	0.001	0.917

*ES* Effect Size, *LDP* Laparoscopic Distal Pancreatectomy, *RDP* Robotic Distal Pancreatectomy, *SD* Standard Difference

^a^Statistically significant

^b^Effect Size is calculated as Theil’s U value for categorical variables and Cohen’s *d* standard difference for continuous variables

**Fig. 3:**
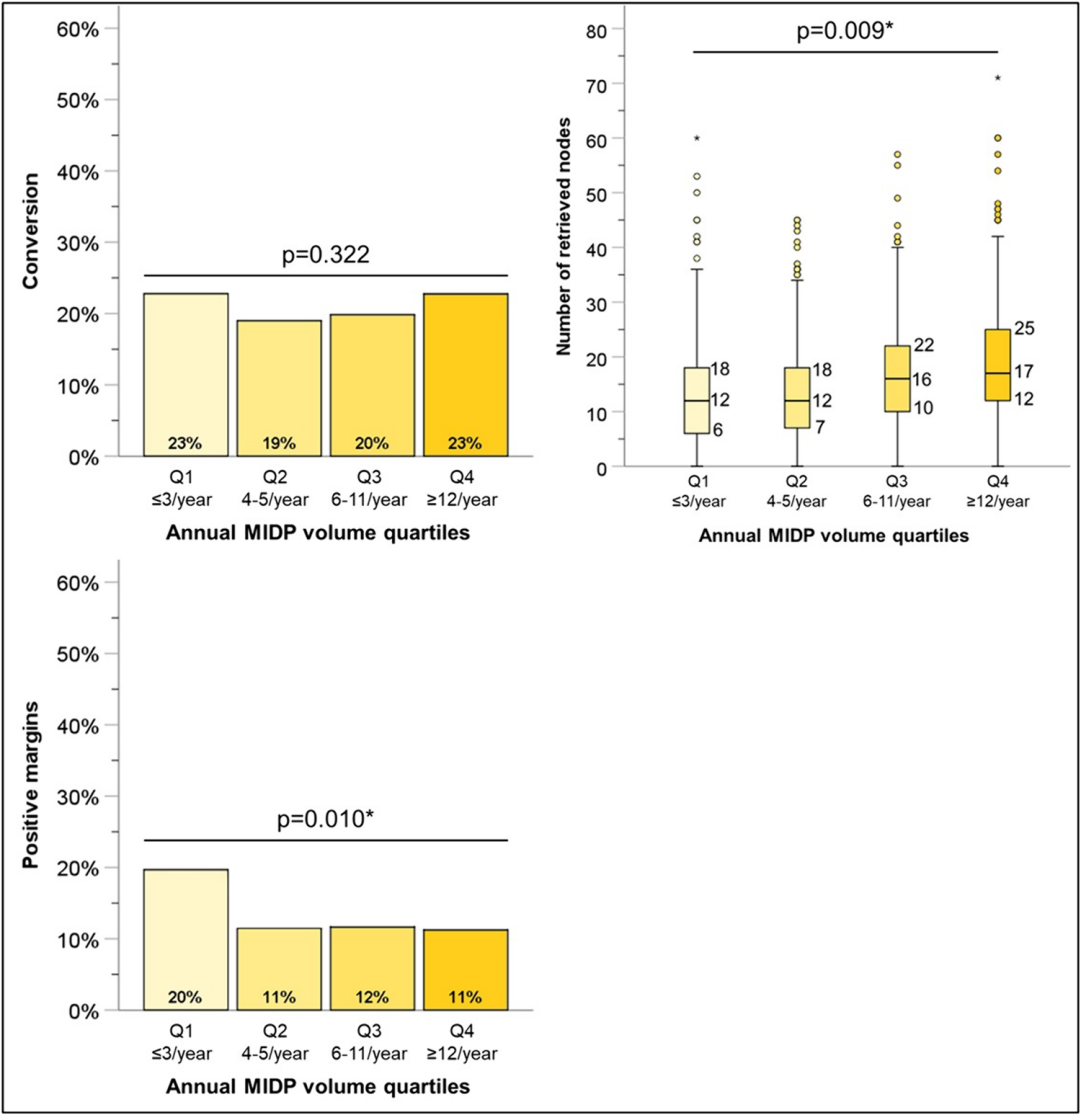
Impact of annual institutional MIDP volume on technical metrics. *MIDP* Minimally Invasive Distal Pancreatectomy. *Statistically significant

**Fig. 4:**
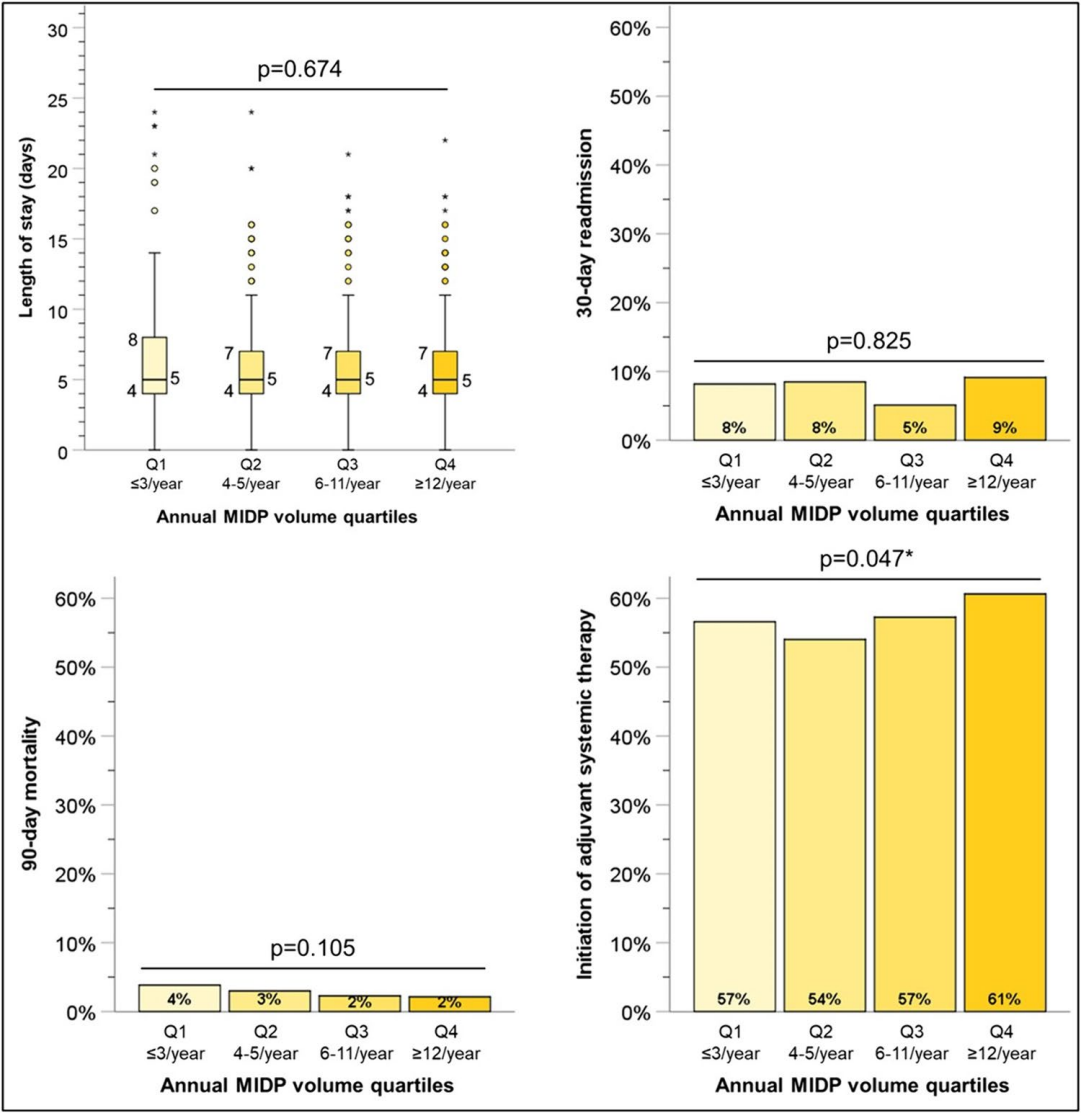
Impact of annual institutional MIDP volume on post-operative metrics. *MIDP* Minimally Invasive Distal Pancreatectomy. *Statistically significant

We next examined the impact of annual case volumes on outcomes of MIDP. There was no difference in the conversion to open rate based on annual MIDP case volume (*P* = 0.32) (Fig. 3). However, there was a difference in the mean node retrieval number (*P* < 0.01) and positive margin rates (*P* = 0.01) between the four determined quartiles. Institutions performing less than three MIDP cases per year retrieved a mean of 12 nodes and had a 20% positive margin rate, while those in the highest quartile (≥ 12 cases per year) retrieved an average of 17 nodes and had a 11% positive margin rate. Comparison of post-operative metrics based on annual case volume quartiles is demonstrated in Fig. 4. Length of stay (*P* = 0.67), 30-day unplanned readmission (*P* = 0.83) and 90-day mortality (*P* = 0.11) demonstrated no difference based on annual case volume quartiles. However, there is a statistically significant difference in the rates of initiation of adjuvant systematic therapy between the quartiles (*P* = 0.05).

## Discussion

This study demonstrates that the national adoption of RDP is rapidly increasing over the past decade. Using propensity matched cases from a national database of hospital-based cohorts, the LDP approach demonstrated nearly a doubled rate of conversion to open, but no difference in short-term morbidity and readmission or technical oncologic outcomes, including initiation of adjuvant systemic therapy after surgery. We further demonstrate that higher volume centers have improved technical oncologic outcomes compared to lower volume centers, even though this bar is low.

Consistent with other studies, there is a higher conversion rate with LDP than RDP that does not translate to a longer length of stay or short-term mortality in our cohort. This may relate to the well-described dexterity available with the RDP approach that is not available with laparoscopic instruments, but may also be attributed to surgeon selection bias for technically straightforward cases. In our unmatched data from Table 2, the robotic cohort had smaller T1 or T2 tumors while the the laparoscopic group were mostly T3 tumors. However, it should be noted that conversion to open surgery, although frequently used as a metric of success for minimally invasive surgery, is not always an indication of technical failure. Rather, conversion to open surgery may demonstrates appropriate intraoperative decision making for safe completion of a procedure for a patient. This is supported by the lack of difference in conversion rate in all MIDP cases based on institution annual case volume.

There have been three recent randomized clinical trials [4, 6, 9] comparing the MIDP to the open approach. All three collectively suggest that MIDP, when performed by trained surgeons, offers an advantage in terms of functional recovery with no significant differences in complication rates, including reduced incidence of pancreatic specific outcomes such as delayed gastric emptying [10]. The DIPLOMA trial supported the non-inferiority of the MIDP approach compared to ODP in oncologic technical rates as well [6].

However, there have been no randomized control trials directly comparing the two minimally invasive approaches, although there are multiple, smaller-volume, retrospective papers that have compared LDP and RDP, including an analysis of NCDB data from 2010–2013. This study was limited to just 99 RDP cases, likely attributable to our finding of increasing utilization in recent years [11]. Our study demonstrates similar results, but with nearly quadruple the number of robotic cases. With the addition of propensity score matching (PSM), we also attempted to reduce the confounding biases that may be in the data. This has been performed on RDP versus LDP groups before, but were limited to single institutions smaller sample sizes that included a variety of benign and malignant indications for surgery [12, 13]. Our study focuses on a cohort of PDAC only and utilizes a national dataset. Our study also included year of the procedure in the PSM as it is a critical variable in the matching model especially for robotic resections since the robotic platform was gradually introduced into the field of pancreas surgery during the inclusion window of the study, as evident in our trends figure. Interestingly, there was a higher rate of the 30-day readmission rate for the RDP group in our PSM analysis not demonstrated in these studies, although there was no difference in mortality or initiation of adjuvant therapy.

Although differences in short-term surgical outcomes were not demonstrated in our study based on institution annual case volume, unfortunately, very low-volume centers which perform ≤ 3 cases per year demonstrated worse technical oncologic outcomes, with a significantly higher rates of positive margins and a lower nodal harvest. Margin status is one of the most important predictors of survival in resectable PDAC [14, 15]. Additionally, while the volume of adequate lymph node harvest is debatable, with a range of 11 to 20 cited in the literature [16–18], thorough lymph node evaluation is critical for a sound oncologic resection. As such, the clinical significance of the difference in the number lymph node harvest for high and low-volume centers cannot be fully elucidated. This high rate of positive margins and lower nodal harvest did not translate into a difference in the short-term mortality of these patients. However, the consequence for survival may be demonstrated in longer term survival outcomes not included in the scope of this study.

Regionalization of pancreatic surgery to only high-volume centers is a focus of debate [19], with possible consequences such as decreasing access to care for patients and super sub-specialization of general surgery. Strengths of utilization of the NCDB is that it includes a variety of practice settings, although most of the cases were performed in academic/research programs in metropolitan areas. Additionally, it is critical to understand that annual institutional surgical volume is not analogous to outcomes. There may be significant variability of outcomes within any hospital system regardless of case volume [20], which may be more pronounced in low-volume hospitals.

In this study, the highest volume centers in our study performed only more than 12 cases per year. One study suggested that the cutoff for high-volume centers should be seven or more DPs per year [21]. Conversely, a recent international, multicenter, retrospective cohort study from Europe established that 85 MIDP cases per surgeon are necessary to obtain a composite primary textbook outcome specific to pancreas surgery [22]. This emphasizes that the case volume of the institution as analyzed in this study is not a substitution for surgeon experience.

Limitations of this study include those inherent to any retrospective study. Of a total of 9357 MIDP cases available in the NCDB, 83.6% of patients were excluded from analysis due to incomplete data, non-PDAC diagnosis, or concurrent resection of multiple organs. There is also intrinsic selection bias for a surgical approach by surgeons that may affect surgical outcomes. An attempt to control for these variables were completed with utilization of propensity score matching using CCI as a proxy for comorbidities and T stage for tumor size. Other considerations in the surgical decision making, such as prior abdominal surgeries, are lacking. In addition, the NCDB does not have pancreas specific surgical outcomes such as blood loss and post-operative pancreatic fistula, which are technically and clinically important considerations for surgeons. As the study focused on case volume of the institution and not the surgeon, the learning curves of the surgeons performing the procedure could not be established within the scope of the study and may confound analysis. Finally, this study was not able to assess long-term oncologic outcomes but utilized short-term surrogates of technical oncologic outcomes, including initiation of adjuvant chemotherapy.

## Conclusion

Laparoscopic and robotic distal pancreatectomy have similar peri- and post-operative surgical and oncologic outcomes, with a higher rate of conversion to open in the laparoscopic cohort. Increased institution case volume is correlated with improved oncologic operative success, re-demonstrating the importance of institution experience in minimally invasive pancreatic surgery.

## Data Availability

The National Cancer Database is a publicly available dataset through the American College of Surgeons that can be requested through instructions on the following link: https://www.facs.org/quality-programs/cancer/ncdb
